# Auricular therapy improves gastrointestinal function in patients with gynecological laparoscopic surgery: A protocol for systematic review and meta analysis

**DOI:** 10.1097/MD.0000000000023421

**Published:** 2020-12-04

**Authors:** Ying Hu, Xianying Cheng, Xinglin Su, Yun Fu

**Affiliations:** a Zhejiang Hospital; b Sandun District Zhejiang Hospital, Hangzhou, Zhejiang Province, China.

**Keywords:** ear therapy, gastrointestinal function, gynecologic laparoscope, meta-analysis, protocol

## Abstract

**Background:**

Gynecological laparoscopic surgery is the main method to treat gynecological diseases, but postoperative gastrointestinal reactions are more common in patients. Auricular therapy, as a characteristic therapy of Traditional Chinese Medicine, can improve gastrointestinal symptoms such as nausea and vomiting by stimulating the conduction of acupoints through the nervous system on internal organs, but there are studies questioning the efficacy of auricular therapy. Therefore, the purpose of this study is to prove the efficacy and safety of auricular therapy in promoting gastrointestinal function recovery after gynecological laparoscopic surgery, and to provide reference value for future clinical practice.

**Methods:**

To search English databases (PubMed, Excerpta Medical Database [Embase], Web of Science, the Cochrane Library) and Chinese databases (Chinese National Knowledge Internet [CNKI], WanFang, Viper, Chinese Biomedical Literature Database) by computer, and conduct a randomized controlled trial on the effect of aural point therapy on gastrointestinal function recovery of patients after gynecological laparoscopic surgery from the establishment of the database to October 2020. Two researchers independently evaluate the quality of the included studies and extract the data, and meta-analysis of the included literature is carried out using RevMan5.3 software.

**Results:**

In this study, the efficacy and safety of auricular therapy in the recovery of gastrointestinal function after gynecological laparoscopic surgery are evaluated from the aspects of first anal exhaust time, bowel sound recovery time, and incidence of gastrointestinal complications.

**Conclusion:**

This study will provide reliable evidence-based evidence for auricular therapy in the treatment of gastrointestinal function after gynecologic laparoscopic surgery.

**Ethics and dissemination:**

Private information from individuals will not be published. This systematic review also does not involve endangering participant rights. Ethical approval was not required. The results may be published in a peer-reviewed journal or disseminated at relevant conferences.

**OSF Registration number:**

DOI 10.17605 / OSF.IO / ZSPGA

## 1 Introduction

With the popularity of laparoscopy in clinical practice, it has become the main means of clinical treatment of gynecological diseases. However, postoperative complications of gastrointestinal tract are more common in patients, usually presenting symptoms such as abdominal distension, defecation and exhaust disorder, nausea, and vomiting.^[1]^ It is currently believed that these symptoms are closely related to intraoperative anesthesia and hyperuricemia caused by carbon dioxide pneumoperitoneum and intestinal damage.^[2]^ In clinic, antihistamines and anticholinergic drugs are commonly used to prevent and improve gastrointestinal symptoms after gynecological laparoscopic surgery, but these drugs may cause different degrees of side effects.^[3]^ Therefore, its application in clinical practice is limited.

Studies^[4]^ suggest that auricular plaster therapy can effectively improve gastrointestinal dysfunction.

Auricular point sticking, as a characteristic therapy of Traditional Chinese Medicine (TCM), is simple and safe in clinical application. Moreover, according to TCM theory, auricular points are closely related to the internal nervous system, and stimulation of corresponding points can affect the body function through the nervous system conduction.^[5]^ Multiple studies at home and abroad^[6–8]^ have proved that auricular point pressing can improve gastrointestinal symptoms after gynecological laparoscopic surgery, reduce the incidence of nausea, vomiting and abdominal distension, and advance the first anal exhaust time.

At present, although several randomized controlled studies have shown that auricular therapy can reduce the incidence of gastrointestinal discomfort after gynecological laparoscopic surgery to a certain extent and the degree of gastrointestinal discomfort.^[9,10]^ However, there are still many studies questioning the effectiveness of auricular therapy,^[11,12]^ and there are differences in the existing clinical trials in terms of research scheme and efficacy evaluation, resulting in uneven research results, which to some extent affects the reliability of research results and the promotion of this therapy. Therefore, this study brings into a randomized controlled trial (RCT) of auricular therapy for gastrointestinal dysfunction after gynecological laparoscopic surgery, to objectively evaluate its effectiveness and safety, and to provide an effective basis for clinical promotion.

## 2 Methods

### 2.1 Protocol register

This protocol of systematic review and meta-analysis has been drafted under the guidance of the preferred reporting items for systematic reviews and meta-analysis protocols (preferred reporting items for systematic reviews and meta-analysis-P). Moreover, it has been registered on the open science framework (OSF) on October 22, 2020. (registration number: DOI 10.17605/OSF.IO/ZSPGA).

### 2.2 Ethics

This study does not refer to patients’ privacy and does not require the approval of the ethics committee or the informed consent of the patients.

### 2.3 Eligibility criteria

#### 2.3.1 Types of studies

We collected all available RCTs on Auricular plaster therapy for gastrointestinal function after gynecological laparoscopic surgery, unlimited in the number of magazines, time of publication, and whether to use blind methods. And the language is limited to Chinese and English.

#### 2.3.2 Research objects

The subjects are women with gynecological laparoscopic surgery, regardless of race, region, or age, excluding patients with other severe organ dysfunction, previous intestinal obstruction or intestinal surgery, and advanced ovarian cancer.

#### 2.3.3 Intervention measures

The treatment group adopts auricular therapy plus conventional nursing. And auricular therapy includes auricular plaster therapy and auricular acupuncture therapy, among which the location and treatment time of auricular acupoint are not limited. The control group is given routine nursing, which includes diet, exercise, and so on.

#### 2.3.4 Outcome indicators

(1) The first anal exhaust time;
(2) Recovery time of bowel sounds;
(3) Incidence of gastrointestinal complications (nausea, vomiting, abdominal distension, and other abdominal discomfort);
(4) Incidence of adverse reactions.

### 2.4 Exclusion criteria

(1) On the basis of auricular point treatment, the treatment group combines with other TCM treatments, such as internal medicine, acupuncture, moxibustion, and so on;
(2) The study of auricular point treatment before operation;
(3) Unable to obtain the full text of the research;
(4) Studies with incorrect data or incomplete information, but still unable to be solved by contacting the author;
(5) Literature with high bias risk assessed by randomization or allocation concealment;^[13]^
(6) For the repeatedly published literature, select the literature with the most comprehensive data and highest quality.

### 2.5 Retrieval strategy

The retrieval method combines subject words and free words. The computer searches Chinese National Knowledge Internet (CNKI), WanFang, Viper, Chinese Biomedical Literature Database, and Chinese search terms are “Fu Qiang Jing (*Laparoscope)”* or “Ti Qiang Jing (*Celioscope*)” or “Fu Qiang Nei Kui Jing (*Abdominal endoscope*)” and “Er Xue (*Auricular point*).” Retrieval in English databases including PubMed, Excerpta Medical Database [EMBASE], Web of Science, the Cochrane Library, Search terms in English are *Perioneoscope*, *Celioscope*, *Acupressure*, *Auricular point shank*, *Ear points*. The retrieval time is from the time of construction to October 2020, and a RCT is collected on the effect of aural point treatment on gastrointestinal tract of patients after gynecological laparoscopic surgery. Take PubMed as an example, and the retrieval strategy is shown in Table 1.

**Table 1:** PubMed database retrieval strategy.

Number	Search terms
#1	Laparoscopes[MeSH] Laparoscopes[Title/Abstract]
#2	Peritoneoscope[Title/Abstract]
#3	Celioscope[Title/Abstract]
#4	#1 OR #2 OR #3
#5	Acupressure[MeSH]
#6	Acupressure[Title/Abstract]
#7	Otopoint[Title/Abstract]
#8	Auricular point sticking[Title/Abstract]
#9	Ear points[Title/Abstract]
#10	Ear acupressure[Title/Abstract]
#11	Ear pressure[Title/Abstract]
#12	Ear pressure beans[Title/Abstract]
#13	Ear hole buried beans[Title/Abstract]
#14	Ear sticking[Title/Abstract]
#15	Ear massage[Title/Abstract]
#16	Ear acupuncture treatment[Title/Abstract]
#17	Ear therapy[Title/Abstract]
#18	Ear buried seed[Title/Abstract]
#19	Ear pressure seeds[Title/Abstract]
#20	#5 OR #6 OR #7 OR #8 OR #9 OR #10 OR #11 OR #12 OR #13 OR #14 OR #15 OR #16 OR #17 OR #18 OR #19
#21	#4 AND #20

### 2.6 Data filtering and extraction

**Figure 1:**
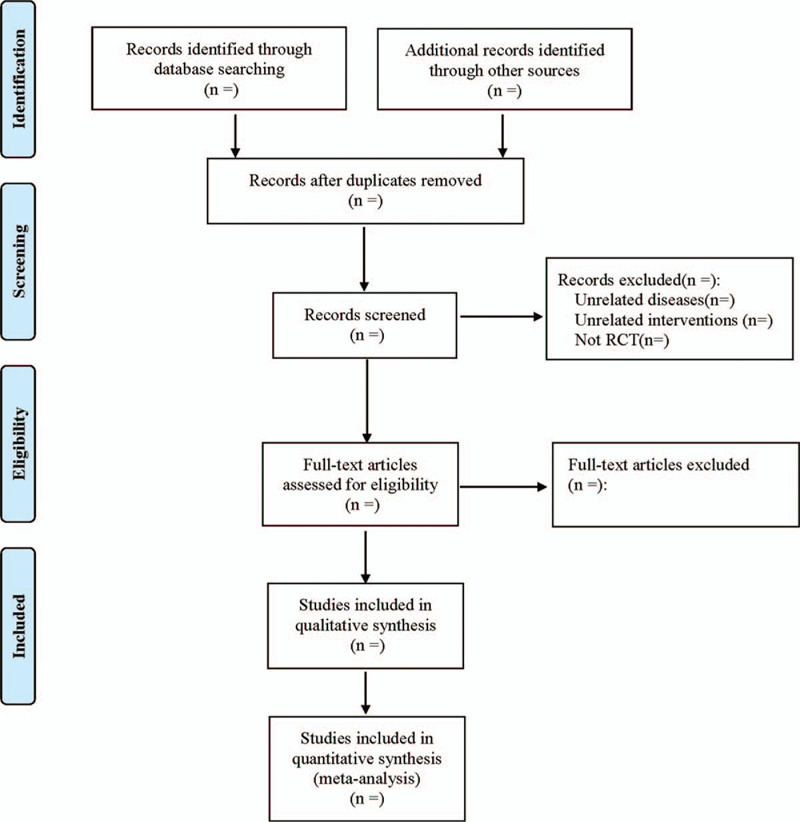
Flow diagram.

By referring to the method of literature screening in the Cochrane Collaboration Handbook of Systematic Reviewers (VERSION 5.0), the two researchers use the EndNoteX7 literature management software to download the literature from various databases. After the review, the literature is selected, and the preliminary screening is conducted according to the title and abstract of the article. Then, the full text is read for further literature screening. Finally, the required literature is included. Two researchers independently screen and extract literature information, and check each other. In case of differences, they could negotiate to solve them or ask a third researcher to assist in judgment. The extraction of data mainly includes the basic information of the included literature, including title, first author's name, year of publication, journal of publication, country where the study is conducted, and so on. Basic information about the subjects includes average age, sex, sample size, ethnicity, and severity. Intervention methods of treatment group and control group include auricular therapy, auricular acupoint, course of treatment, etc. Information about outcome measures. Information on literature quality evaluation includes random grouping method, allocation, and hiding, blind method, etc. The process of literature filtering is shown in Figure 1.

### 2.7 Literature quality evaluation

Literature bias risk assessment is conducted using the built-in risk bias assessment tool of ReviewManager5.3 software (the Cochrane collaboration's tool for assessing risk of bias). The evaluation includes the following 7 parts:

(1) The generation of random sequences;
(2) The allocation of random schemes is hidden;
(3) The subjects of the study and the implementers of the intervention are blinded;
(4) The evaluator was blinded;
(5) Completeness of outcome indicator data;
(6) Selective reporting;
(7) Other aspects of bias.

The 2 researchers shall make a judgment of low risk, unclear and high risk for the above items, and cross-check after completion, respectively. In case of any disagreement, discussion shall be conducted. If no agreement can be reached, discussion shall be made with the researchers of the third party.

### 2.8 Statistical analysis

RevMan5.3 software is used for meta-analysis. Dichotomous variables are expressed by relative ratio. The measurement data is presented as the weighted mean difference if the measurement tool is identical to the measurement unit; and if the measurement tool or unit is inconsistent, the standard mean difference is used as the effect size. Heterogeneity between the included study results is analyzed by *χ*^2^ test, test level α equals to 0.05, the value of *I*^2^ is used to judge the heterogeneity. If *P* ≥ .1, I^2^≤50% indicates low heterogeneity, a fixed-effect model is adopted. If *P* < .1, *I*^2^ > 50% indicates significant inter-study heterogeneity, the source of heterogeneity should be analyzed. In the absence of significant clinical and methodological heterogeneity, random-effects models are used for analysis. If there are obvious clinical heterogeneity and methodological heterogeneity, methods such as subgroup analysis or sensitivity analysis will be used; if the clinical heterogeneity is too obvious and subgroup analysis cannot be performed, only descriptive analysis will be conducted.

#### 2.8.1 Dealing with missing data

If there is missing data in the article, contact the author via email for additional information. If the author cannot be contacted, or the author has lost relevant data, descriptive analysis will be conducted instead of conducting meta-analysis.

#### 2.8.2 Subgroup analysis

According to the difference of auricular therapy, the treatment group can be divided into two subgroups: auricular plaster therapy group and auricular acupuncture therapy group. According to the age of the patients, they can be divided into 3 subgroups: young group, middle-aged group, and old group. Subgroup analysis is carried out according to different gynecological diseases. Subgroup analysis is performed according to the course of treatment.

#### 2.8.3 Sensitivity analysis

To determine the stability of outcome indicators, sensitivity analysis is used to analyze each outcome indicator.

#### 2.8.4 Assessment of reporting biases

Funnel plots will be used to assess publication bias if no fewer than 10 studies are included in an outcome measure. In addition, Egger and Begg tests are used for the evaluation of potential publication bias.

## 3 Discussion

Gynecological laparoscopic surgery has been widely used in clinical practice due to its advantages of less trauma and faster recovery. But after laparoscopic surgery, patients will have a high probability of complications, including more gastrointestinal reactions.^[14]^ Patients usually have nausea, vomiting, abdominal distension, and other symptoms, which are closely related to anesthesia during surgery and hormone secretion in the patient's body. Studies have shown that increased plasma motilin levels in patients after abdominal surgery are the main physiological factors that contribute to gastrointestinal dysfunction.^[15]^ At the same time, a number of studies have confirmed that different anesthesia methods have different effects on the gastrointestinal function of patients after laparoscopic surgery. CO_2_ pneumoperitoneum at different intraoperative pressures causes different degrees of damage to the peritoneum and other abdominal tissues of patients, which further causes abdominal distension, nausea and vomiting, and other symptoms in patients.^[16,17]^ For the treatment of gastrointestinal complications in patients after laparoscopic surgery, drug intervention is commonly used clinically. Although the gastrointestinal discomfort symptoms of patients can be alleviated to a certain extent, most drugs have side effects and adverse reactions, and their long-term effects have not been proved by evidence-based medicine.^[18,19]^

Auricular therapy, as a TCM method, is simple, safe, and has few adverse reactions. And it is widely used in clinic.^[20]^ A large quantity of studies^[21,22]^ has confirmed that it can effectively regulate gastrointestinal function, which is related to the anatomical basis of auricular points. The auricular branch of the vagus nerve is the only branch on the body surface of the vagus nerve that innervates gastrointestinal function. Auricular sticking can improve gastrointestinal motility by stimulating the vagus nerve.^[23]^ TCM believes that different areas on the auricle correspond to different parts of the five viscera and 6 organs of the human body, so stimulating the acupoints of related areas of the gastrointestinal tract can regulate the gastrointestinal function. Studies^[24]^ showed that auricular point sticking and pressing on both sides of the large intestine, stomach, and spleen after operation could relieve abdominal distension after operation of uterine fibroids. Physiologically, auricular point sticking stimulates the vagus nerve, regulates the autonomic nerve, inhibits the release of plasma motilin, and thus improves gastrointestinal function.^[22,23]^

Recent research has found that auricular point sticking has obvious effect in the treatment of gastrointestinal dysfunction after gynecological laparoscopic surgery. Most of the studies started the intervention at 6 hours after the operation, and the pressure was pressed once every two hours for 2 minutes at a time. Meanwhile, some studies combined auricular point pressing with other exercise methods to treat the patients, and all of them saw significant improvement in indicators such as the first postoperative exhaust time and the recovery time of bowel sounds.^[21,25]^ Studies^[21]^ suggested that auricular point pressing could regulate the level of plasma motilin and regulate the autonomic nerve, thus improving the gastrointestinal symptoms of patients. However, its clinical efficacy has not been recognized by international authoritative medical organizations. Therefore, it is necessary to carry out systematic evaluation and meta-analysis on RCT of auricular plaster therapy for gastrointestinal function after gynecological laparoscopic surgery, so as to objectively evaluate its efficacy and provide objective evidence for clinical application. This study also has some limitations: this included article only involves Chinese and English literature, and ignores studies in other languages. At the same time, the methods of auricular compatibility in different literatures are different, and the gynecological diseases of patients are also different. There is some clinical heterogeneity among studies, so more high-quality studies are needed to confirm the efficacy of auricular therapy on gastrointestinal dysfunction after gynecological laparoscopic surgery.

## Author contributions

**Data curation:** Ying Hu, Xianying Cheng.

**Funding acquisition:** Yun Fu.

**Investigation:** Ying Hu, Xianying Cheng.

**Literature retrieval:** Ying Hu and Xianying Cheng.

**Software:** Xinglin Su.

**Supervision:** Yun Fu.

**Writing – original draft:** Ying Hu, Xianying Cheng.

**Writing – review & editing:** Ying Hu, Yun Fu.
